# A Trial-Based Cost-Utility Analysis of Metastasis-Directed Therapy for Oligorecurrent Prostate Cancer

**DOI:** 10.3390/cancers12010132

**Published:** 2020-01-04

**Authors:** Elise De Bleser, Ruben Willems, Karel Decaestecker, Lieven Annemans, Aurélie De Bruycker, Valérie Fonteyne, Nicolaas Lumen, Filip Ameye, Ignace Billiet, Steven Joniau, Gert De Meerleer, Piet Ost, Renée Bultijnck

**Affiliations:** 1 Department of Urology, Ghent University Hospital, 9000 Ghent, Belgium; karel.decaestecker@uzgent.be (K.D.); Nicolaas.lumen@uzgent.be (N.L.); 2 Department of Human Structure and Repair, Ghent University, 9000 Ghent, Belgium; valerie.fonteyne@uzgent.be (V.F.); gert.demeerleer@uzleuven.be (G.D.M.); piet.ost@ugent.be (P.O.); renee.bultijnck@ugent.be (R.B.); 3 Department of Public Health and Primary Care, Ghent University, 9000 Ghent, Belgium; ruben.willems@ugent.be (R.W.); lieven.annemans@ugent.be (L.A.); 4 Department of Radiotherapy and Experimental Cancer Research, Ghent University Hospital, 9000 Ghent, Belgium; aurelie.debruycker@uzgent.be; 5 Department of Urology, AZ Maria Middelares, 9000 Ghent, Belgium; filip.ameye@azmmsj.be; 6 Department of Urology, AZ Groeninge, 8500 Kortrijk, Belgium; Ignace.billiet@azgroeninge.be; 7 Department of Urology, UZ Leuven, 3000 Leuven, Belgium; steven.joniau@uzleuven.be; 8 Department of Radiotherapy, UZ Leuven, 3000 Leuven, Belgium; 9 Faculty of Medicine, University of Ghent, 9000 Ghent, Belgium; 10 Research Foundation-Flanders (FWO), 1000 Brussels, Belgium

**Keywords:** cost-utility analysis, metastasis-directed therapy, oligorecurrent, prostate cancer, oligometastasis, prostatic neoplasms, cost-effective, markov model

## Abstract

The optimal management of patients with oligorecurrent prostate cancer (PCa) is unknown. There is growing interest in metastasis-directed therapy (MDT) for this population. The objective was to assess cost-utility from a Belgian healthcare payer’s perspective of MDT and delayed androgen deprivation therapy (ADT) in comparison with surveillance and delayed ADT, and with immediate ADT. A Markov decision-analytic trial-based model was developed, projecting the results over a 5-year time horizon with one-month cycles. Clinical data were derived from the STOMP trial and literature. Treatment costs were derived from official government documents. Probabilistic sensitivity analyses showed that MDT is cost-effective compared to surveillance (ICER: €8393/quality adjusted life year (QALY)) and immediate ADT (dominant strategy). The ICER is most sensitive to utilities in the different health states and the first month MDT cost. At a willingness-to-pay threshold of €40,000 per QALY, the cost of the first month MDT should not exceed €8136 to be cost-effective compared to surveillance. The Markov-model suggests that MDT for oligorecurrent PCa is potentially cost-effective in comparison with surveillance and delayed ADT, and in comparison with immediate ADT.

## 1. Introduction

Oligorecurrent prostate cancer (PCa) is hypothesized to be an intermediate state of PCa with a restricted metastatic capacity [1]. According to the European Association of Urology (EAU) guidelines, the treatment options for these patients are independent of the number of metastases and consist of immediate or delayed androgen-deprivation therapy (ADT) [2]. There is a growing interest in treating oligorecurrent patients with a metastasis-directed approach aiming to postpone disease progression and the need for definitive, palliative ADT. Metastasis-directed therapy (MDT) by means of surgery or radiotherapy has already been widely evaluated in several retrospective analyses [3,4]. To date, only one prospective randomized trial, the Surveillance or Metastasis-Directed Therapy for Oligometastatic Prostate Cancer Recurrence (STOMP) trial, evaluated the effect of MDT versus surveillance in PCa [5], where MDT was either performed via surgery or via stereotactic body radiotherapy (SBRT) to the metastatic lesion(s). The median ADT-free survival after MDT was 21 months (80% confidence interval (CI): 14–29), which was better compared to 13 months (80% CI: 12–17) for patients on surveillance with delayed ADT (HR 0.6, 80% CI 0.4–0.9, *p* = 0.11). Therefore, the primary aim was to assess the cost-utility of MDT (either by means of surgery or SBRT) versus surveillance in Belgium, based on the STOMP trial [5]. Secondly, since immediate ADT is described in the guidelines as well [2], as a subset of this study, immediate ADT versus MDT was investigated.

## 2. Results

### 2.1. Base Case

In the base case analysis with a 5-year time horizon and an average patient age of 68 years, total mean discounted cost and discounted QALY per patient were respectively €17,088 and 3.86 for MDT, €15,673 and 3.74 for surveillance and €21,145 and 3.41 for ADT. The discounted ICER for MDT vs. surveillance was €11,374. For MDT vs. ADT, the mean gain in discounted cost for MDT was €4058 and the mean gain in QALY was 0.45.

### 2.2. Probabilistic Sensitivity Analyses for Base Case

**Figure 1:**
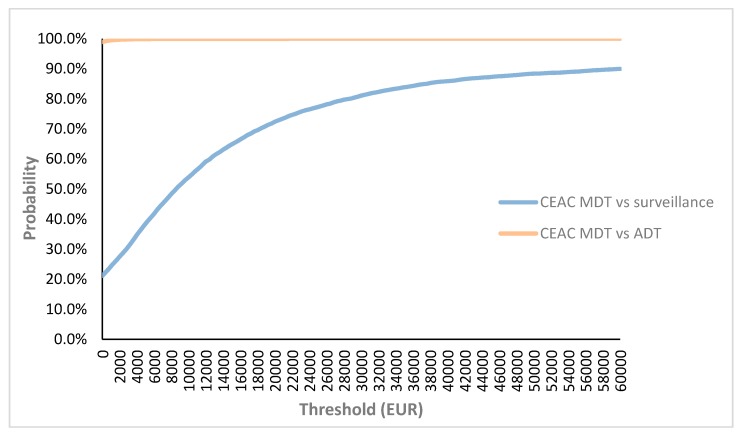
Probabilistic sensitivity analysis-cost-effectiveness acceptability curve. Please remark that the patients in the different groups (i.e., MDT, surveillance and ADT) entered different health states during follow-up and thus the corresponding costs and utilities of that health state were applied. Abbreviations CEAC: Cost-effectiveness acceptability curve; MDT: Metastasis-directed therapy.

**Figure 2:**
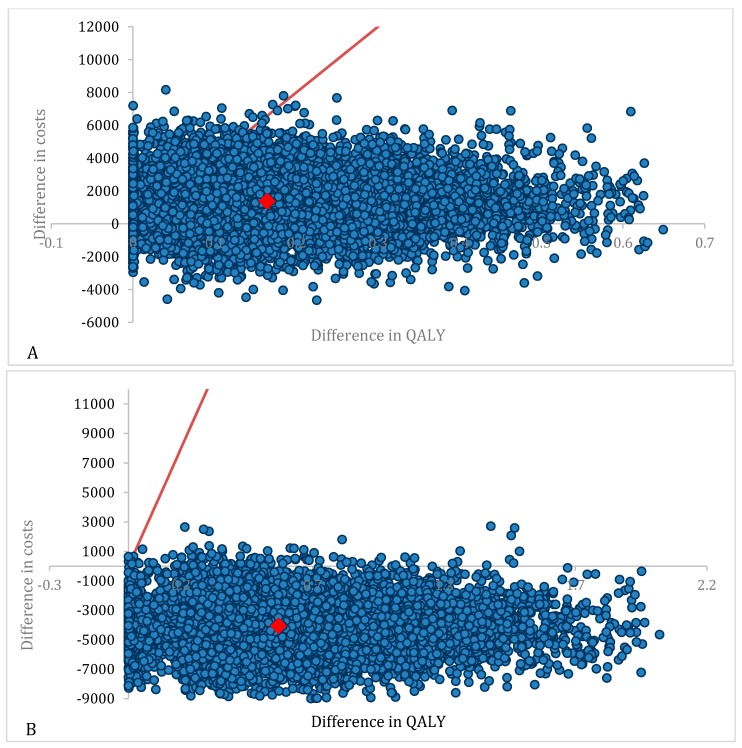
Graphic representation of 10,000 simulations of the cost-utility analysis of MDT versus surveillance. Every dot represents a simulation of the cost-utility analysis. The red line represents the WTP threshold. The red dot represents the mean ICER. Differences are calculated as MDT minus surveillance/ADT. (**A**) Cost-utility plane of MDT versus surveillance. (**B**) Cost-utility plane of MDT versus ADT. Please remark the different scales in Figure 1A,B. Abbreviations; MDT: Metastasis-directed therapy; QALY: Quality adjusted life years; WTP: Willingness-to-pay.

**Figure 3:**
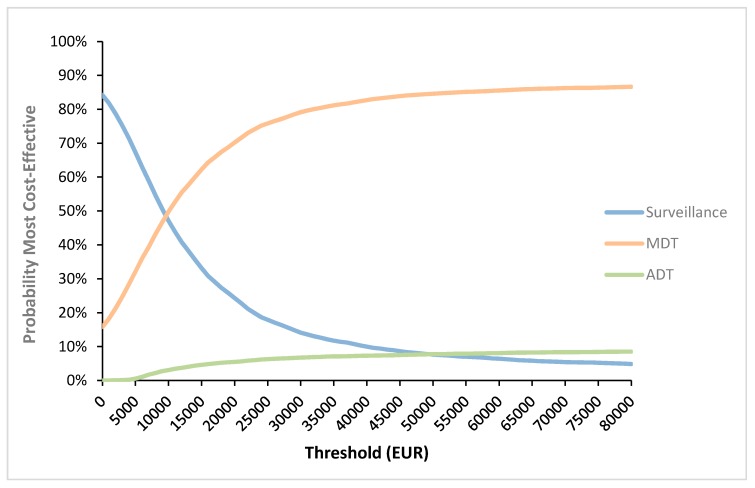
Multiple cost-effectiveness acceptability curve. This curve shows the most cost-effective strategy depending on the willingness-to-pay threshold. Please remark that the patients in the different groups (i.e., MDT, surveillance and ADT) entered different health states during follow-up and thus the corresponding costs and utilities of that health state were applied. ADT: Androgen-deprivation therapy; MDT: Metastasis-directed therapy.

MDT vs. surveillance appeared to result in an 85.9% probability of being cost-effective (Figure 1) with a mean ICER of €8393/QALY (Figure 2A). MDT vs. ADT appeared to be cost-effective in 100% of all iterations (Figure 1) with 99% of the cases being dominant. For the simulations situated in the right upper quadrant (gain in QALY’s but more expensive treatment) the mean ICER was €1065/QALY (Figure 2B). The CEAC showed that, of the three treatment options, MDT has the highest probability of being cost-effective if the WTP threshold exceeds €10,000 (Figure 3).

### 2.3. One-Way Sensitivity Analysis

**Figure 4:**
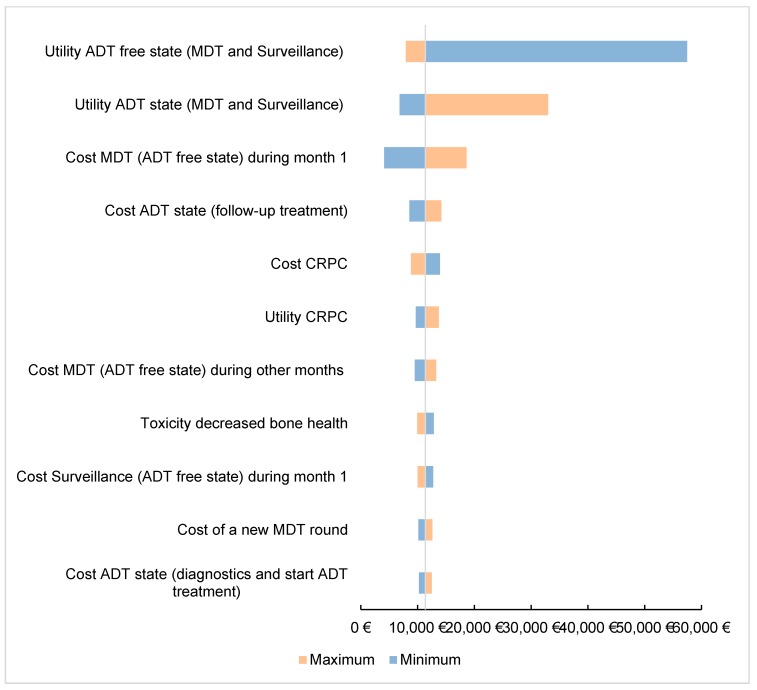
One-way sensitivity analysis—tornado diagram. Tornado model showing the impact of the different variables on the ICER. In this figure, all sensitivity scores were set at 100%. The figure depicts the impact on the ICER when the sensitivity score ranges from 80% to 120%. ADT: Androgen-deprivation therapy; MDT: Metastasis-directed therapy.

Further assessing the robustness of the model, one-way deterministic sensitivity analysis was performed and graphically represented in a Tornado plot (Figure 4). The sensitivity analysis showed that the ICER was mainly sensitive to utility scores of the health states, the cost of MDT, surveillance and CRPC and new round of MDT. Nonetheless, even with the most sensitive parameters, the ICER is still cost-effective, except for a utility score of the ADT-free state of 80% of the base case value. We investigated all input parameters (Appendix A Table A1) that could influence the analysis. The parameters that had no impact on the ICER are not displayed in Figure 4.

### 2.4. Scenario Analyses

**Figure 5:**
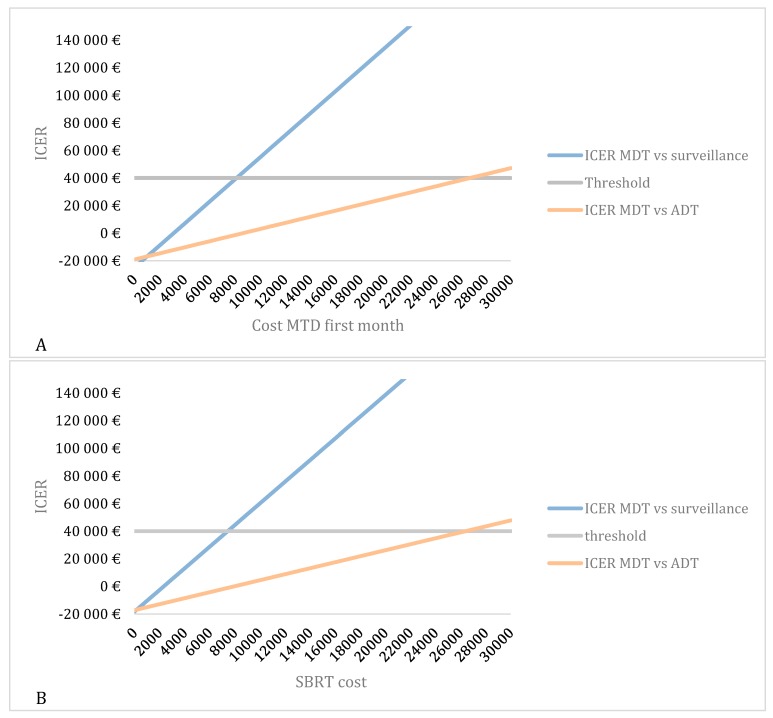
Scenario analyses. (**A**) Overall overview of the cost-utility of MDT versus surveillance or MDT versus ADT in function of the cost of MDT during the first month. (**B**) Overall overview of the cost-utility of MDT versus Surveillance or MDT versus ADT in function of the cost of MDT for SBRT. ADT: Androgen-deprivation therapy; ICER: Incremental cost-effectiveness ratio; MDT: Metastasis-directed therapy; SBRT: Stereotactic body radiotherapy.

Figure 5A gives a graphic representation of the changing cost of MDT and Figure 5B of SBRT alone. As seen in Figure 5A, when comparing MDT vs. surveillance, the cost of MDT can increase up to €8136 for the ICER to remain cost-effective. When comparing MDT with ADT, this cost can even increase until €26,749 until reaching the €40,000 threshold. When looking at the SBRT cost in particular (Figure 5B), the cost of SBRT can increase up to €7435 in order to remain cost-effective compared to surveillance. Compared to ADT this cost can even increase to €26,389.

**Figure 6:**
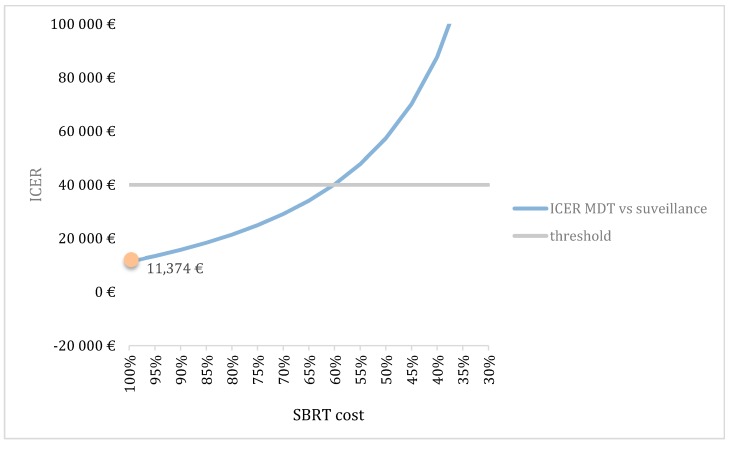
Scenario analysis investigating ICER in function of the effect of MDT versus surveillance. 100% is the effect as it was observed in the STOMP trial (orange dot). As seen in the figure, the ICER becomes higher when the difference in the effect of MDT versus surveillance becomes smaller. ICER: Incremental cost-effectiveness ratio; MDT: Metastasis-directed therapy.

Figure 6 shows the third scenario analysis where the clinical effect of MDT was investigated. In this scenario, we gradually decreased the effect size from 100%, which represents the ICER as observed in the STOMP trial, to a zero effect. MDT appeared to be a cost-effective treatment strategy as long as the prolonged ADT-free survival is at least 60% of the effect observed in the STOMP trial.

**Figure A1:**
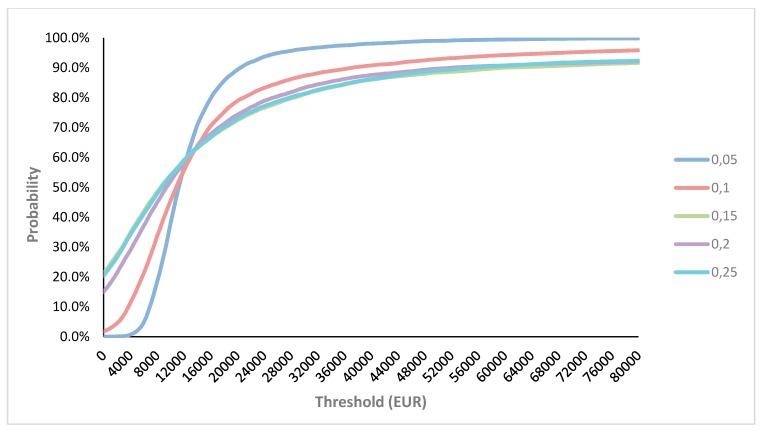
Cost-effectiveness acceptability curve of MDT versus surveillance. In order to evaluate the uncertainty of the input variables, the standard error of the mean was varied: mean * magnitude, with magnitude 0.05, 0.1, 0.15, 0.20, and 0.25. The results were robust, with MDT versus surveillance being cost-effective at a willingness-to-pay threshold of 40,000 euro in respectively 98%, 90.7%, 85.9%, 87.6%, and 86.2% of the iterations. MDT: Metastasis-directed therapy.

In a last scenario analysis we tried to investigate the influence of the standard error by varying the magnitudes of the standard error. However, varying the standard error did not impact the obtained results to a great extend (Appendix A Figure A1).

## 3. Discussion

This study investigated the cost-effectiveness of MDT in oligorecurrent PCa from a Belgian healthcare payer’s perspective. Currently, the use of MDT in oligorecurrent PCa is limited with the STOMP trial being the first randomized controlled trial demonstrating a benefit in ADT-free survival of MDT [5]. Recently, Palma et al. [6] published their results on the use of palliative standard of care systemic therapy alone compared to palliative systemic therapy in combination with SBRT in different primary tumors, with PCa being the most common tumor in the SBRT group (21%). This study showed an overall survival benefit for systemic therapy plus SBRT, however, the PCa group was too small to draw conclusions for this patient population.

To the best of our knowledge this is the first cost-effectiveness study of MDT versus surveillance, in Europe. In absence of an official WTP-threshold in Belgium, the threshold was set at €40,000/QALY [7]. Our results suggest that MDT is potentially cost-effective in oligorecurrent PCa when compared to surveillance. As there were no grade 2 or higher toxicities observed in the STOMP trial, theoretical toxicities were extracted from the literature and incorporated in the analysis. The POPSTAR trial [8] confirmed that the majority of side effects were grade 1 which were not associated with an economic impact. In this trial, only one grade 3 toxicity was observed, being a vertebral fracture. The used dose in this trial (20 Gy in one fraction) differed from that used in the STOMP trial (30 Gy in three fractions). In contrast, the toxicities reported in the recently published SABR-COMET trial [6] were of greater importance. This trial is to date the largest randomized trial comparing SBRT + standard of care versus standard of care alone, showing an improvement in overall survival and progression-free survival in the combination arm which was accompanied by a significant increase in toxicity. The most important side effects were fatigue, dyspnea and pain (grade 2 and 3) and three treatment-related deaths occurred. Nevertheless, it is important to state that the primary tumor in the SABR-COMET trial was PCa in only 21% of the patients treated with the combination. The treatment-related deaths were associated with SBRT on a lung lesion (two cases) and an adrenal metastasis (one case), two locations rather unlikely to arise from PCa [9].

In this analysis, the cost of the CRPC-state was included, however, the cost of the toxicities associated with the treatment of CRPC was not taken into account to facilitate the analysis and because the cost of CRPC was beyond the scope of this manuscript. Please remark that this is associated with an even higher benefit in real life.

Our results show the importance of the value of the WTP threshold of €40,000 per QALY. This threshold was based on the threshold for reimbursement of medicines. As seen in Figure 3, the cost-effectiveness of MDT compared to surveillance varies depending on the applied threshold and becomes cost-effective when a threshold of minimum €10,000 is used. It is important to note that this threshold varies amongst countries, also within the European Union. The WTP threshold of €40,000 per QALY is rather low compared to other countries such as the United States where a WTP threshold of $100,000–150,000 has been suggested [10]. Another important issue are the variables included in the study that might influence cost-effectiveness. Figure 4 shows the most important factors influencing the results. As seen in the figure, the utility of the ADT-free state has the largest impact on the ICER and is the only variable making the ICER non-favorable. The lower this utility, the higher the ICER becomes. When implying a utility that is 80% of the chosen utility of 0.92, the ICER even exceeds the €40,000 per QALY threshold. However, it is unlikely that this utility is of great influence as the probabilistic sensitivity analysis remains cost-effective in almost 86% of iterations, when all parameters are varied (Figure 2A). Although the cost of MDT could be an important influencing factor, even with a cost increase up to 120%, the ICER remains cost-effective. This stresses the importance of the cost of MDT, and in particular SBRT, on the cost-effectiveness. As for the WTP threshold, the cost of SBRT tends to vary across countries. As seen in Figure 5B, the cost of SBRT cannot exceed €7435 for the analysis to be cost-effective.

As per EAU guidelines [2], the recommended treatment of patients presenting with recurrent PCa is immediate or delayed ADT. As the timing of ADT is still under debate, we conducted the same analysis for cost-effectiveness of MDT versus immediate ADT [11]. Since ADT is known to have an important impact on QoL and is associated with greater costs, the ICER of MDT versus ADT is even more favorable compared to MDT versus surveillance. As seen in Figure 5B, the cost of SBRT can increase up to €26,389 to remain cost-effective.

Although this analysis was based on a trial with relatively short available follow-up, MDT appears to be cost-effective compared to surveillance. Important to note is the higher utility score of the ADT-free state (0.92) compared to the ADT state (0.78) which has an important influence on the ICER (Figure 4). As ADT is associated with important side effects, affecting quality of life and higher costs, deferred start of ADT in the MDT group has an important effect on the ICER. Even more, the more patients that the start of ADT in an earlier phase, the more patients that enter the CRPC-state, leading to even higher costs.

The results of this trial suggest that MDT is a cost-effective strategy and it is economically ethical to further explore the long-term results of MDT in phase 3 trials. Besides SBRT, elective nodal radiotherapy (ENRT) is being investigated as a treatment option in these patients. The GETUG P07 trial (Salvage Radiotherapy Combined With Hormonotherapy in Oligometastatic Pelvic Node Relapses of Prostate Cancer-OLIGOPELVIS2-NCT03630666) is a prospective phase 2 trial investigating the role of ENRT as an MDT treatment strategy. The PEACE V study (Salvage Treatment of OligoRecurrent Nodal Prostate Cancer Metastases-STORM-NCT03569241) is a randomized phase 2 trial investigating PLND/SBRT versus ENRT. These trials will provide new data on the role of MDT (radiotherapy and metastasectomy) in oligorecurrent PCa and form the basis of further randomized investigations.

### Limitations

Inevitably, this study was associated with several limitations. First, due to the restrictions of the trial, follow-up was rather short and currently no long-term data are available for these patients. Therefore, time horizon was set at 5 years to limit extrapolation of unknown follow-up data. Second, since this study was trial based, this gave rise to a per-study protocol patient care. We based our results on the current available international guidelines [2], where possible. However, since this is still an investigational treatment option, which is not yet included in the guidelines, no standard follow-up is available. As a consequence of the protocol-based analysis, it is assumable that the entered costs in this analysis were an overestimation. Third, patient groups of the STOMP trial were rather small. Therefore, we opted to only use the transition probabilities of the STOMP trial and use literature for toxicities and QoL data. Fourth, probabilities and health state utilities were extracted from literature and are not always well examined. Health economic evaluations of stage 2 trials are characterized with the adaption of some assumptions. It is recommended to include, for example, quality of life measurements in stage 3 and 4 clinical trials. Nevertheless, this is the first cost-effectiveness analysis of MDT versus surveillance or ADT in Europe. Even when taken these limitations into account, the ICER is situated far below the WTP threshold.

## 4. Material and Methods

### 4.1. Patients and Procedures

Patients included in the STOMP trial [5] presented with a PSA relapse and a limited number (≤3) of nodal (N1/M1a/N1 + M1a), bone (M1b) or visceral (M1c) metastases diagnosed on choline positron emission tomography computed tomography (PET-CT). At inclusion, patients were randomly assigned to surveillance or MDT. In the surveillance group (n = 31), patients did not receive active treatment but were clinically re-evaluated and had a serum PSA control every 3 months, with choline PET-CT at specific PSA thresholds [5]. In total, 31 patients were treated by means of MDT and then followed according to the same protocol as the surveillance group. Surgery was used in five patients with nodal disease (robot-assisted pelvic lymph node dissection (PLND)) and one patient with a lung metastasis (metastasectomy). No patients were operated with an open or laparoscopic approach. All other patients in the MDT arm were treated by means of SBRT (n = 25) [5].

### 4.2. Model Structure

**Figure 7:**
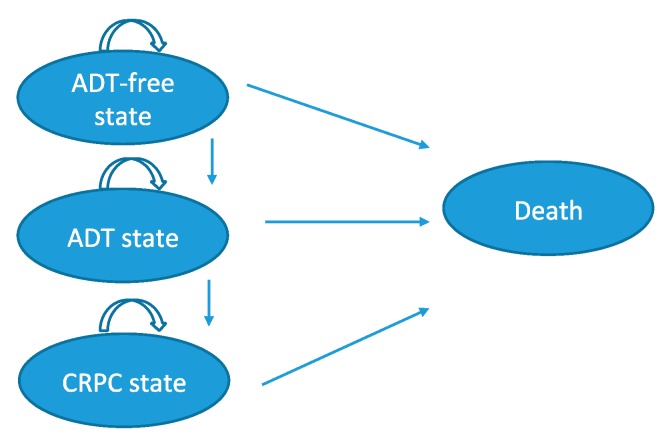
Markov model summarizing the state transitions (simplified model). Patients treated by MDT or surveillance enter the Markov model in the ADT-free state. Patients that are treated with immediate ADT enter the Markov model in the ADT state. Circles represents the different health states in the model. Arrows represent transitions between health states, Patients are at each health state at risk for developing side effects. ADT: Androgen-deprivation therapy; CRPC: Castration-resistant prostate cancer; MDT: Metastasis-directed therapy.

A cost-utility (CU) analysis, which compared MDT with delayed ADT vs. surveillance with delayed ADT, was conducted from a healthcare payer’s perspective. A second comparison was performed between MDT and ADT. A Markov trial-based model can be consulted in Figure 7. A detailed description of the used input variables to build the analysis can be found in Appendix A Table A1 [3,5,9,12,13,14,15,16,17,18,19,20,21,22,23,24,25,26,27,28]. An overview of the used costs per intervention can be found in Appendix A Table A2.

The Markov model included several health states. The first state was the ADT-free state which was defined as each cycle where patients did not require ADT. The second state was the ADT-state where ADT was initiated as a consequence of symptomatic progression, progression to polymetastatic disease (>3 metastases) or local progression of the baseline-detected metastases. The third state was castration-resistant prostate cancer (CRPC)-state where patients developed CRPC as defined by the EAU guidelines [2]. The fourth state was death from all causes. At the end of each cycle, patients could remain in the current health state or transit to another state. Possible multiple rounds of MDT were included in the analyses, based on the STOMP data. All costs associated with ADT in the MDT or surveillance group are included in the analyses. The comparator in the STOMP trial was surveillance, however, since according to the guidelines, standard of care for these patients is systemic treatment with ADT, a third treatment arm has been added [2]. Patients receiving immediate ADT entered the model in the ADT-state. The model was programmed in Microsoft^®^ Excel. 

### 4.3. Time Horizon

A 5-year time horizon with one-month cycles and half-cycle corrections has been modeled. Median follow-up in the STOMP trial was three years. An extrapolation was made up to 5 years for ADT-free survival rates. A longer time horizon is difficult to justify as there are no data available regarding the health state transition probabilities and would be associated with more uncertainty.

### 4.4. Discounting

Future costs and benefits were discounted at a yearly rate of 3% and 1.5%, respectively, as recommended for health economic evaluations in Belgium [22].

### 4.5. Model Inputs

Model parameters for our trial-based analysis were derived from the STOMP study by Ost et al. [5] and the current medical literature. Model inputs are described below, and an overview of the model inputs can be found in the Appendix A Table A1 [3,5,9,15,16,17,18,20,21,22,23,26,28].

#### 4.5.1. Health State Transition Probabilities

Transition probabilities were extracted from the study by Ost et al. [5], distributed equally over six-month periods. The transition probability for ADT-state to CRPC-state was extracted from De Bruycker et al. [9].

#### 4.5.2. Non-Prostate Cancer-Specific Mortality

Death due to other causes, is defined as death due to all causes other than PCa. Death from other causes was based on 2017 age-specific life tables for males in Belgium [23] and was adjusted for PCa death [24]. The risk of dying from PCa was only taken into account in the CRPC-state and was extracted from the study of De Bruycker et al. [9].

### 4.6. Toxicities

Toxicities of grade 2 or higher were considered as having a clinical and economic significant impact on the model. In the STOMP trial [5], 17% of patients presented with grade 1 toxicity for MDT. No grade 2 or higher toxicity was observed. Based on literature [3,25,26,27] and expert opinion, a list of possible grade 2 or higher toxicities were used for the model MDT toxicities.

In contrast to MDT, toxicities of ADT are well described in the literature. The study by Walker et al. [28] and the international EAU-ESTRO-ESUR-SIOG guidelines on PCa [12] were used for the ADT side effects. The review by Nguyen et al. [13] was used to define the evidence-based strategies per side effect and the study by Bultijnck et al. [14] was used to incorporate the intervention rate per side effect in clinical practice. The cost of the possible side-effects of ADT was integrated in the model. The toxicity cost of next line systemic drugs in CRPC setting was not taken into account since this was beyond the scope of this study.

### 4.7. Utilities

Health outcomes were presented using QALYs to incorporate both the quantity (number of life years) and the quality (utility score) of life. A utility value ranges from 0 to 1, where 0 represents death and 1 perfect health. Each of the health states were assigned with a utility weight corresponding with the intervention. Quality of life data was not extracted from the STOMP trial because of the limited available data. Therefore utility scores were obtained from the literature [15,16,17,18] using the cost-effectiveness analysis registry to find the individual studies [19]. As we used different sources, the chosen values were expert opinion and sensitivity analyses were performed. Utility scores differ for SBRT and surgery, therefore the ratio of SBRT and surgery usage in the STOMP trial was taken into account.

### 4.8. Costs

The healthcare payer’s perspective has been applied since employment rates were expected to be negligible in the target population (average age of 68 year) and other patient costs (e.g., transportation) were excluded. The perspective included both costs for the national healthcare insurance and the patient co-payment. All costs are expressed in Euro (€) and were extracted in 2018. Treatment costs were obtained from the Belgium National Institute for health and disability insurance (*Rijksinstituut voor ziekte- en invaliditeitsverzekering*, RIZIV) database [20] and cross-checked from hospital invoices from the Ghent University Hospital. Unit costs for drugs were based on the official listings of the Belgian center for pharmacotherapeutic information [21]. There are different possible ADT drugs available, however, the least expensive drug was chosen in the analysis since level 1 evidence for a better outcome with a specific type of ADT is still lacking [2] (range of ADT drug costs in Belgium per month: 63.4–141.5 €) [21]. Treatment costs included the cost of diagnostics, initial treatment and follow-up costs. A full list per health state can be found in Appendix A Table A2. All calculations included the possibility of multiple rounds of SBRT, based on data of the STOMP trial [5].

### 4.9. Statistical Analysis

The main outcome was the incremental cost-effectiveness ratio (ICER), which was calculated as follows:

$$ICER=\frac{\mathrm{COS}\mathrm{Tmdt}-\mathrm{COS}\mathrm{Tsurveillance}}{*\mathrm{QALYmdt}-\mathrm{QALYsurveillance}}$$

* Quality-adjusted life years (QALY) is a measure of quantity (number of life years) and the quality of life.

A treatment strategy with an ICER less than the societal willingness-to-pay (WTP) threshold is cost-effective, and a negative ICER means that the new strategy generates more health at a lower long-term cost (i.e., dominant strategy). In Belgium, a WTP has not been formalized so the threshold is set at €40,000 per QALY. This is in line with the reimbursement threshold often cited for medications [29].

*One-way sensitivity analysis* was performed to identify key parameters impacting the ICER. The low and high end of each parameter were set on 80% and 120% of the deterministic value, respectively. This was to evaluate the uncertainty of the chosen analysis.

In addition, all parameters included in the one-way sensitivity analysis were varied simultaneously in a second order Monte-Carlo simulation (i.e., probabilistic sensitivity analysis). Costs were modeled using a gamma distribution, and probabilities and utilities were modeled using a beta distribution [30]. Ten thousand iterations of input parameters were randomly sampled. A maximum limit was accounted for in the probabilistic analysis with the assumption that a more progressed disease state always has a lower utility score than the previous one (e.g., CRPC-state utility cannot be higher than ADT-state utility).

In the analysis, MDT was compared with surveillance. A second analysis compared MDT with immediate ADT. For completeness, a multiple cost-effectiveness acceptability curve (CEAC) visualized the most cost-effective treatment option at varying thresholds.

*Scenario analyses* were conducted to determine the impact of input parameters with different scenarios. The SBRT cost and first month MDT cost were varied in two different scenarios to determine the maximum cost price for still being cost-effective at a €40,000/QALY threshold. Furthermore, we conducted a scenario analysis to predict the cost-effectiveness in case of a non-significant difference in ADT-free survival of MDT versus surveillance. A last scenario analysis was conducted to take into account the uncertainty around the standard error of the literature, data scenario analysis was performed on these standard errors (resp. 0.05, 0.1, 0.15, 0.2 and 0.25).

## 5. Conclusions

This trial-based cost-utility study suggests that MDT is potentially cost-effective in the treatment of oligorecurrent PCa patients compared to surveillance with delayed ADT and even to immediate ADT. However, this analysis was based on the results of the STOMP trial which had a phase 2 design. Nevertheless, this study suggests a health economic advantage of MDT and supports ongoing phase III studies to confirm its efficacy. When more (follow-up) data are available, it is recommended to provide an update of the model.

## Appendix A

**Table A1:** General overview of the model input variables.

Inputparameter	Deterministic Value	Standard Error	Distribution	Source
Mean age (years)	68.15	NA	NA	[5]
Discount rate costs (%)	3%	NA	NA	[22]
Discount rate utilities (%)	1.5%	NA	NA	[22]
Disease-related probabilities (%)				
ADT-free survival in MDT	6 months: 94%	NA	NA	[5]
	12 months: 67%			
	18 months: 46%			
	24 months: 46%			
ADT-free survival in surveillance	6 months: 85%	NA	NA	[5]
	12 months: 56%			
	18 months: 32%			
	24 months: 23%			
Transition to CRPC state from ADT state	6 months: 1%	NA	NA	[9]
	12 months: 7%			
	18 months: 12%			
	24 months: 20%			
	30 months: 26%			
	36 months: 28%			
	42 months: 32%			
	48 months: 35%			
Mortality risk				
Death from other causes	68 year: 0.018123	NA	NA	[23]
	69 year 0.019817			
	70 years: 0.020578			
	71 years 0.023583			
	72 years 0.026009			
PCa death in CPRC state	5.7143%	NA	NA	[9]
*Utilities* *				
MDT during the first month	0.72	0.17	Beta	[15]
ADT-free state	0.92	0.23	Beta	[16,17]
ADT state	0.78	0.19	Beta	[17]
CRPC state (chemotherapy accounted for 30% in this state)	0.6	0.15	Beta	[16,18]
*Costs intervention (€)*				
MDT cost month 1 (ADT-free state)	4549	1137	Gamma	[20,21]
MDT cost other months (ADT-free state)	47	12	Gamma	[20]
Surveillance cost month 1 (ADT-free state)	865	216.3	Gamma	[20]
Surveillance cost other months (ADT free state)	17	4.4	Gamma	[20]
Cost diagnostics and start ADT (ADT state)	1266	317	Gamma	[20,21]
Cost ADT in follow-up months ADT (ADT state)	298	74.7	Gamma	[20,21]
CRPC costs (combination of diagnostics, treatment and follow-up) (distribution of abiraterone acetate 35%, enzalutamide 35% and docetaxel 30%)	775	193	Gamma	[20,21]
ADT toxicities probabilities				
Gynecomastia	13%	NA	Beta	[28]
Osteoporosis	10%	NA	Beta	[28]
Diabetes	9%	NA	Beta	[28]
Fatigue	80%	NA	Beta	[28]
Sexual dysfunction	95%	NA	Beta	[28]
Reduced penile/testis size	93%	NA	Beta	[28]
Hot flashes	80%	NA	Beta	[28]
Cognitive changes	48%	NA	Beta	[28]
Anemia	13%	NA	Beta	[28]
Metabolic syndrome	55%	NA	Beta	[28]
MDT toxicities probabilities				
Lymph oedema	0.4%	0.001	Beta	[3]
Anemia needing blood transfusion	0.2%	0.0005	Beta	[3]
Symptomatic lymphocoele	5%	0.0125	Beta	[3]
Neuropraxia	0.4%	0.001	Beta	[3]
Pain	1%	0.0025	Beta	Expert opinion
Diarrhea	4%	0.01	Beta	[26]

* An average utility per health state (and thus treatment) was used, rather than a disutility per possible side effect. ADT: Androgen-deprivation therapy; CRPC: Castration-resistant prostate cancer; MDT: Metastasis-directed therapy; NA: Not applicable; PCa: Prostate cancer.

**Table A2:** Detailed overview of the cost.

Cost	Specification	Cost Estimates (€)
Cost at diagnosis	Imaging (choline PET CT, MR soft tissue and MR total spine), consultation, laboratory monitoring and multidisciplinary oncological consultation (MOC)	Choline PET-CT: 747.72 MR: 207.48 Consultation at urologist/radiation oncologist: 25.43 PSA laboratory: 30.5 MOC: 61.54
Initial treatment-SBRT	Physician fees, CT-simulation, planning, treatment, drugs, laboratory monitoring (with calculated possibility of new SBRT round)	SBRT: 3782.24
Initial treatment-ADT	We investigated the different sorts of ADT (Luteinizing hormone releasing hormone (LHRH)- agonist and antagonist), taken into account the frequency of injection, the associated consultation, etc. and decided to use the drug associated with the lowest cost	Range of costs of different ADT: 63.40–141.5 per month
Initial treatment-surgery	Physician fees, anesthetic drugs, hospital admission, medication and laboratory monitoring	Robot-assisted PLND: 3109.35
Cost of surveillance group	Diagnostics cost, follow-up visit and laboratory monitoring	Choline PET-CT: 747.72 MR: 207.48 Consultation at urologist/radiation oncologist: 25.43 PSA laboratory: 30.5
Treatment CRPC state	Diagnostics costs (imaging, consultation, laboratory and MOC), three possible treatment strategies (abiraterone acetate (AA), enzalutamide and docetaxel *), monitoring costs depending on the treatment.	CRPC first month inclusive diagnostic costs and ADT: 1165.76 Overall mean cost of CRPC state per month: 775.66

Costs are expressed in Euro (€) and were extracted in 2018 [20,21] * Based on expert opinion, distribution of the treatments was respectively set at 35, 35 and 30%. AA: abiraterone acetate; ADT: Androgen-deprivation therapy; CRPC: Castration-resistant prostate cancer; MDT: Metastasis-directed therapy; NA: Not applicable; PCa: Prostate cancer; QALY: Quality-adjusted life years.
